# Neuroimmune Communication in the Kidney

**DOI:** 10.31662/jmaj.2020-0024

**Published:** 2020-07-08

**Authors:** Yasuna Nakamura, Tsuyoshi Inoue

**Affiliations:** 1 Division of Nephrology and Endocrinology, The University of Tokyo Graduate School of Medicine, Tokyo, Japan; 2 Division of CKD Pathophysiology, The University of Tokyo Graduate School of Medicine, Tokyo, Japan

**Keywords:** neuroimmune interaction, Acute kidney injury, Cholinergic anti-inflammatory pathway, vagus nerve stimulation

## Abstract

Recent studies have clarified the interaction between nervous systems and immunity regarding the manner in which local inflammation is regulated and systemic homeostasis is maintained. The cholinergic anti-inflammatory pathway (CAP) is a neuroimmune pathway activated by vagus nerve stimulation. Following afferent vagus nerve stimulation, signals are transmitted to immune cells in the spleen, including β2-adrenergic receptor-positive CD4-positive T cells and α7 nicotinic acetylcholine receptor-expressing macrophages. These immune cells release the neurotransmitters norepinephrine and acetylcholine, inducing a series of reactions that reduce proinflammatory cytokines, relieving inflammation. CAP contributes to various inflammatory diseases such as endotoxemia, rheumatoid arthritis, and inflammatory bowel disease. Moreover, emerging studies have revealed that vagus nerve stimulation ameliorates kidney damage in an animal model of acute kidney injury. These studies suggest that the link between the nervous system and kidneys is associated with the pathophysiology of kidney injury. Here, we review the current knowledge of the neuroimmune circuit and kidney disease, as well as potential for therapeutic strategies based on this knowledge for treating kidney disease in clinical settings.

## Introduction

Acute kidney injury (AKI) and chronic kidney disease (CKD) are highly prevalent diseases with high mortality and morbidity. Although this creates a large national healthcare burden, few strategies have been implemented to stop the progression of these diseases. Inflammation is one of the key factors that mediates the progress kidney injury. Recent experimental studies revealed that AKI and the immune system are closely linked during the damage and recovery stages ^(1)^. Studies of neuroimmune interactions have clarified the linkage between immune circuits and peripheral organs ^(2)^, as well as the pathophysiology of inflammatory diseases, cancer, autism, and multiple sclerosis. The cholinergic anti-inflammatory pathway (CAP) is a well-established inflammatory reflex that occurs via autonomic nerves and neurotransmitters. In this circuit, vagus nerve stimulation has been shown to be effective for mitigating inflammation.

Growing evidence suggests that CAP activation has renoprotective effects in animal models. In this review, we focus on the immune system and kidney disease and summarize current knowledge related to vagus nerve stimulation for therapeutic applications.

## Kidney Injury and Inflammation

**Figure 1.**
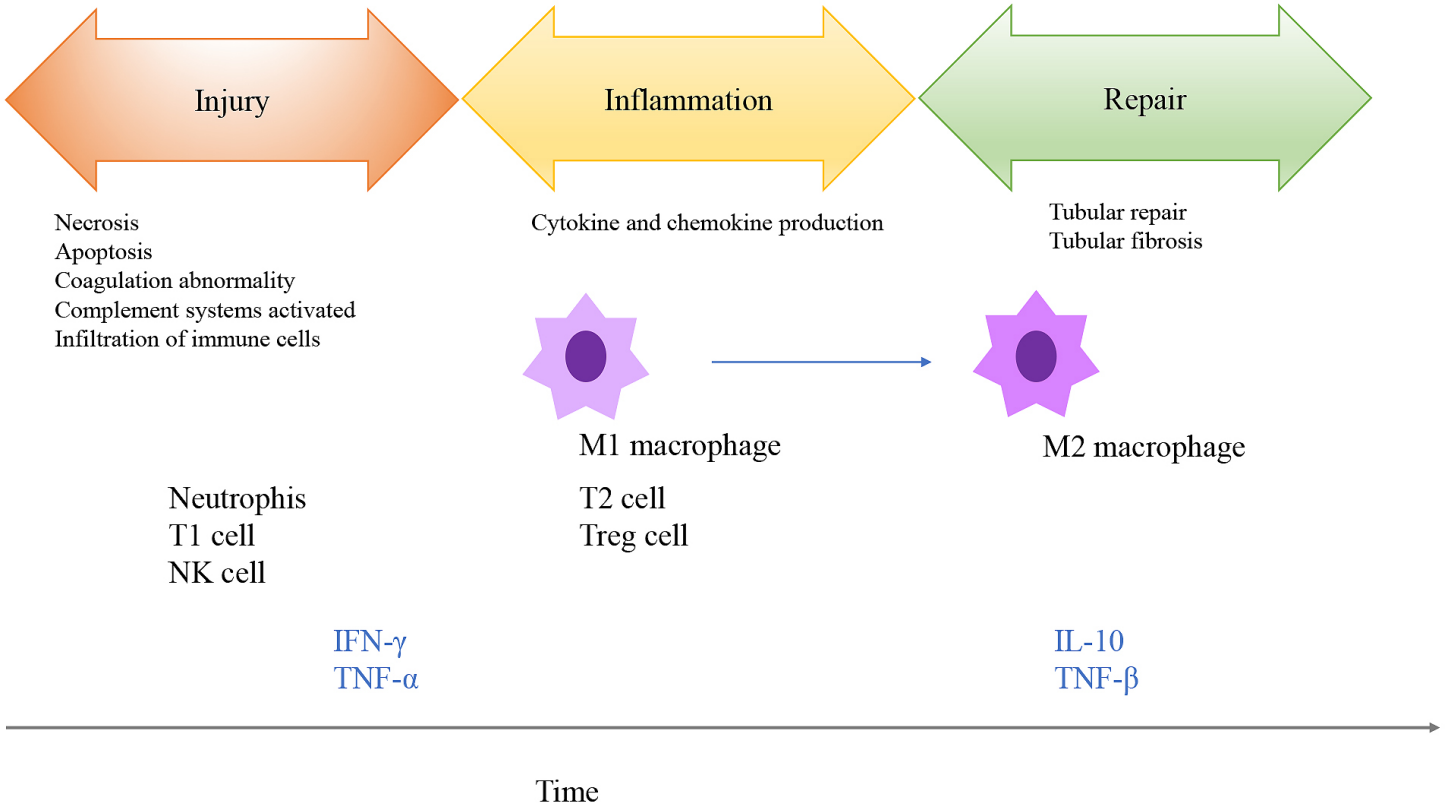
Immune response in ischemia-reperfusion injury kidney. Both innate and adoptive immune systems are involved in ischemia-reperfusion injury model. Kidney injury occurred according to the timeline. Immune system activated in post-ischemic kidneys by resident dendritic cells and macrophages. Disruption of endothelial cells causes an influx of immune cells to damaged kidneys. Abbreviations: NK cell; natural killer cell, Treg cell; regulatory T cell.

Ischemia, nephrotoxic agents, and sepsis are major causes of AKI. For CKD, oxidative stress, hypoxia, and inflammation are the main factors leading to renal fibrosis and tubular damage. Inflammation is considered one of the causes of kidney injury in both AKI and CKD ^(3)^. Ischemia-reperfusion injury (IRI) is an experimental model that mimics aseptic AKI, and the immune system is involved in the inflammatory process caused by ischemia (Figure 1) ^(4)^. The healthy kidney contains few resident immune cells such as dendritic cells, macrophages, and mast cells to maintain homeostasis. IRI causes inflammation in the outer medulla, which is the part of the kidney with the lowest oxygen tension, leading to epithelial cell necrosis. IRI subsequently induces renal vascular epithelial integrity, increases vascular permeability, and promotes infiltration of immune cells into the ischemic kidney. Next, damage-associated molecular patterns released from dying cells, adhesion molecules, hypoxia-inducible factors, and Toll-like receptors induce the recruitment of various immune cells into the injured kidney in the early phases (Figure 1) ^(1)^. These immune cells function in a time-dependent manner. Neutrophils, natural killer cells, and natural killer T cells are recruited to the kidney within hours of ischemia; these immune cells release proinflammatory molecules, such as interleukin-1, interleukin-6, and tumor necrosis factor (TNF)-α. Natural killer cells directly kill tubular epithelial cells, contributing to late-phase damage. Complement systems, proinflammatory cytokines, and chemokine production induce leukocyte infiltration into post-ischemic regions in the kidney ^(5)^. Dendritic cells increase after injury to mediate inflammation. Regulatory T cells exert renoprotective effects and prompt tubular regeneration, mainly in the late phase ^(6), (7)^. B-cell deficiency in mice confers renal protection in the early phase of renal IRI. B cells also traffic into post-ischemic kidneys and differentiate into plasma cells during the repair phase of IRI. Macrophages are key immune cells in ischemic kidneys. In the early phase of IRI, inflammatory monocytes are recruited to the damaged kidney and differentiate into macrophages. These mature macrophages are polarized into M1 (proinflammatory) macrophages and enhance tissue damage. In contrast, in the late phase, these cells are mainly converted to M2 (anti-inflammatory) macrophages which repair tissue damage ^(8), (9), (10), (11)^.

## Neuroimmune Interactions

**Figure 2.**
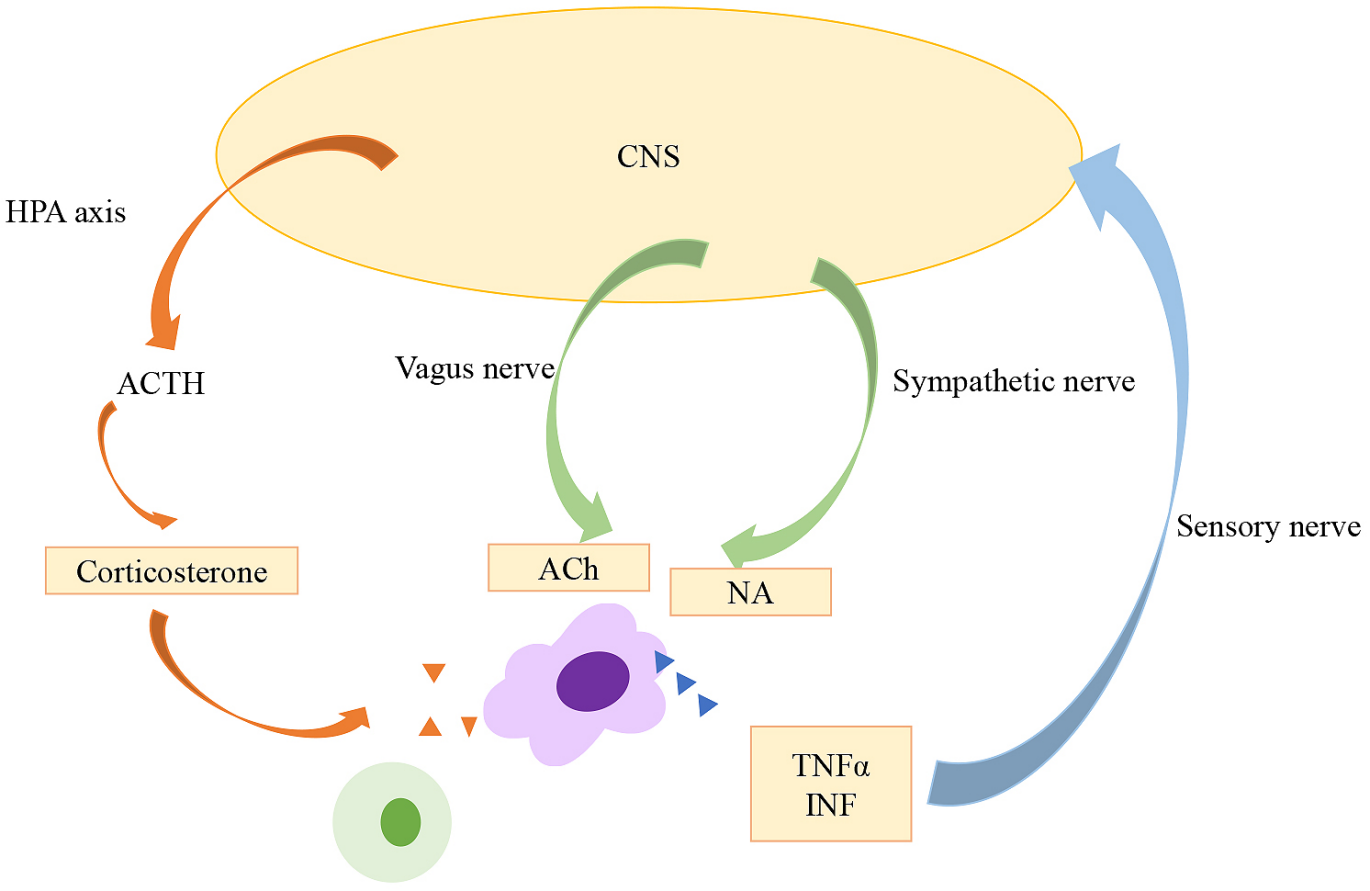
Inflammatory pathway. There are several pathways involved in inflammatory response. HPA axis and sympathetic/parasympathetic nerves are linked. Abbreviations: CNS; central nervous system, HPA axis; hypothalamic-pituitary-adrenal axis, ACTH; adrenocorticotrophic hormone, ACh; acetylcholine, NA; noradrenaline.

Neuroscience and immunology were recognized as separate fields of study until the latter half of the 20^th^ century, when studies showed that some inflammatory responses are regulated by the nervous system. The hypothalamic-pituitary-adrenal (HPA) axis is a stress adaptation response mediated by glucocorticoids (Figure 2). The HPA axis is regulated by the sympathetic, parasympathetic, and limbic circuits innervating the paraventricular nucleus of the hypothalamus ^(12)^. Once secretory neurons of the paraventricular nucleus are stimulated, the HPA axis is activated and evokes a series of reactions; corticotropin-releasing hormone is released from the anterior pituitary grand, after which adrenocorticotrophic hormone is released by pituitary corticotrophs, and systemic release of glucocorticoid from adrenal cells is induced. Besedovsky et al. reported that local inflammation altered signaling by the hypothalamus ^(13)^. In the 1980s, studies suggested that immunity and neuroendocrine systems interact via signaling ligands and receptors ^(14)^. Breder et al. detected interleukin-1 and TNF-α, known as inflammatory cytokines, in the brain following inflammatory reactions ^(15), (16)^. These reactions are considered bi-directional, as cytokines can activate the HPA axis, and immune cells can produce neuropeptides and neurotransmitters.

## Cholinergic Anti-inflammatory Pathway (CAP)

**Figure 3.**
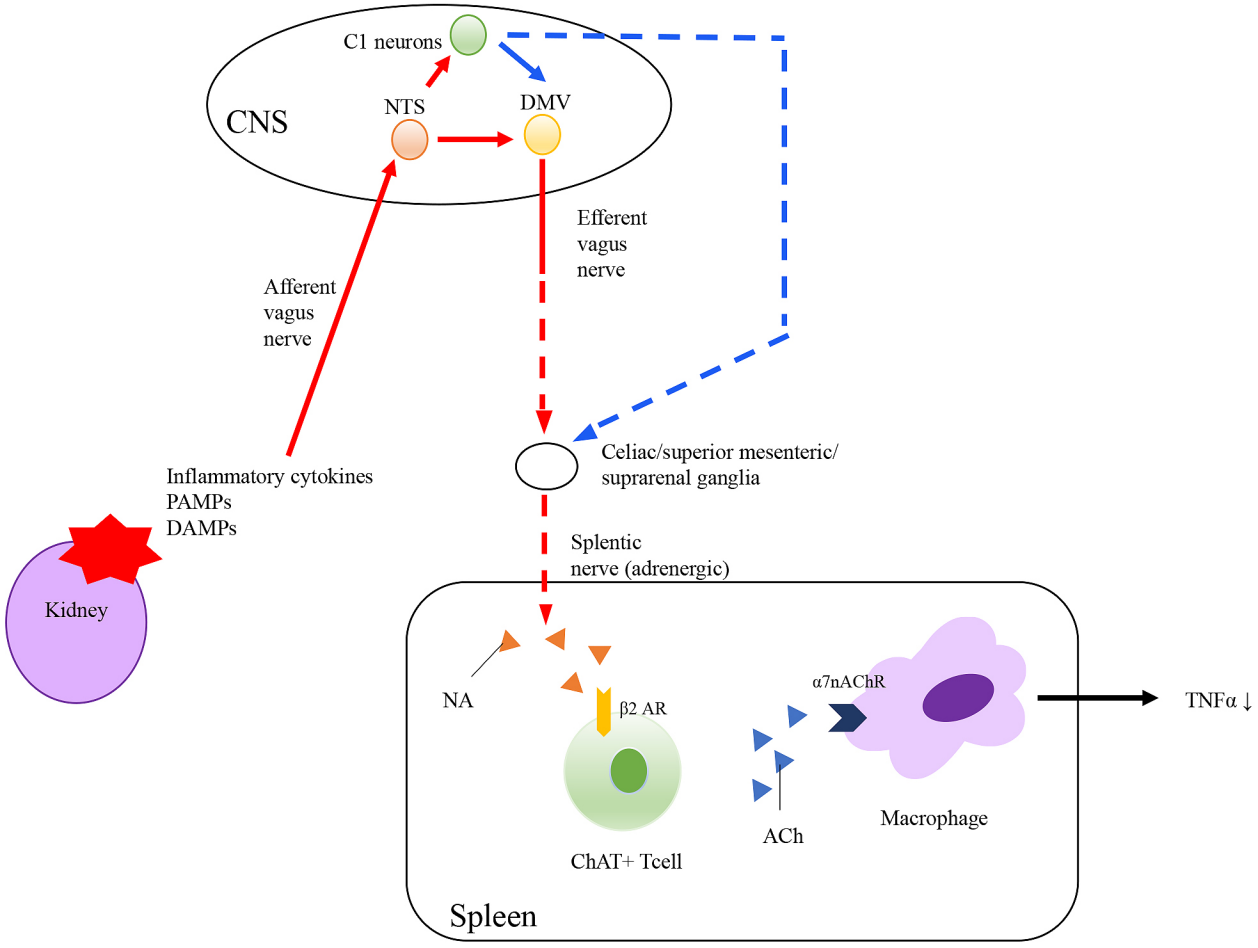
Overview of cholinergic anti-inflammatory pathway (CAP). Once local inflammation produces proinflammatory cytokines, afferent vagus nerves detect these inflammatory signals and transmit them to NTS to DMV in medulla oblongata. These signals are transferred to the spleen via efferent vagus nerve (unknown). Noradrenaline released from the terminal of splenic nerve, binds to β2 adrenergic receptors in the CD4 positive ChAT T cells. Acetylcholine released from these cells binds to α7 nicotinic receptors in macrophages, resulting in decrease in inflammatory responses. Abbreviations: CNS; central nervous system, NTS; nucleus tractus solitarius, DMV: dorsal motor nucleus of the vagus, NA; noradrenaline, ACh; acetylcholine, ChAT; choline acetyltransferase, PAMPs; pathogen-associated molecular patterns, DAMPs; damage-associated molecular patterns.

In 2000, Tracey and colleagues demonstrated that efferent vagus nerve stimulation reduced TNF-α synthesis and septic shock in lipopolysaccharide (LPS)-induced endotoxemia mice ^(17)^. This response was prevented under efferent vagus nerve vagotomy, and thus, they considered this phenomenon as a reflex that occurred via the vagus nerve. They termed this series of reactions the “cholinergic anti-inflammatory pathway” (CAP) (Figure 3) ^(18)^. CAP activation begins with the release of inflammatory molecules (such as pathogen-associated molecular patterns, damage-associated molecular patterns, proinflammatory cytokines, immunoglobulins, and ATP) from damaged tissues; these molecules bind to the receptors of vagal afferent nerves ^(19), (20), (21), (22), (23), (24), (25)^. The signals transmit to the nucleus tractus solotarius in the brainstem ^(17)^, followed by immune cells of the spleen through afferent fibers. The afferent tracts mechanism remains unknown. The alpha 7 subunit of the nicotinic acetylcholine receptor (α7nAChR)-positive macrophages and CD4-positive T cells having β2-adrenergic receptor (β2AR) in the spleen are key mediators of CAP. 

Borovikova et al. demonstrated that efferent vagus nerve stimulation reduced LPS-induced TNF-α elevation, and this response occurred via acetylcholine ^(17)^. This reduction in TNF-α was prevented in α7nAChR knockout mice ^(26)^. Additionally, nicotinic receptor agonists (acetylcholine and nicotine) suppress LPS-induced TNF-α production in peritoneal macrophages, which was abolished in peritoneal macrophages from α7nAChR knockout mice ^(26)^. These results suggest that α7nAChR on macrophages is important for CAP. Following α7nAChR activation, inhibition of the nuclear translocation of nuclear factor (NF)-κB and activation of the JAK2-STAT3 pathway result in reductions in the levels of inflammatory mediators.

Splenectomy was performed in mice to investigate the relationship between the reticuloendothelial systems and CAP. TNF-α production by LPS is suppressed by vagus nerve stimulation, but this effect was abolished in mice that underwent prior splenectomy ^(27)^. CD4-positive T cells also play important roles in CAP. Vida et al. demonstrated that vagus nerve stimulation did not decrease serum TNF-α levels in β2AR knockout mice and T lymphocyte-deficient nude mice ^(28)^. Furthermore, β2AR agonists inhibit cytokine production in the spleen and prevent systemic inflammation in wild-type mice but not in β2AR knockout mice. Transfer of CD4-positive CD25-negative T cells to both β2AR knockout mice and nude mice reestablished the anti-inflammatory effects of vagus nerve stimulation. 

Although the spleen is crucial for CAP activation, the source of acetylcholine in the spleen remained unclear. The efferent fiber of vagus nerve is cholinergic, whereas the splenic nerve is adrenergic, which affects CAP activation ^(29)^. Rosas-Ballina et al. revealed that choline acetyltransferase (ChAT)-expressing CD4-positive T cells, which consist primarily of memory T cells (CD44-high and CD62L-low), are important sources of acetylcholine ^(30)^. The reduction in TNF-α following vagus nerve stimulation was abolished in nude mice, which are devoid of functional T cells. In contrast, adoptive transfer of ChAT-expressing CD4-positive T cells into nude mice partially restored the reduction of TNF-α. This suggests that vagus nerve stimulation requires ChAT-expressing CD4-positive T cells to reduce TNF-α in endotoxemia.

Cumulatively, these studies showed that β2-adrenergic receptor positive-CD4-positive T cells and α7nAChR-positive macrophages in the spleen are crucial for the anti-inflammatory effect of vagus nerve stimulation. However, the mechanism of the release of norepinephrine from the spleen (via the vagus nerve, splenic nerve, or other signal transduction pathways) remains unknown.

## Innervation in the Kidney

**Figure 4.**
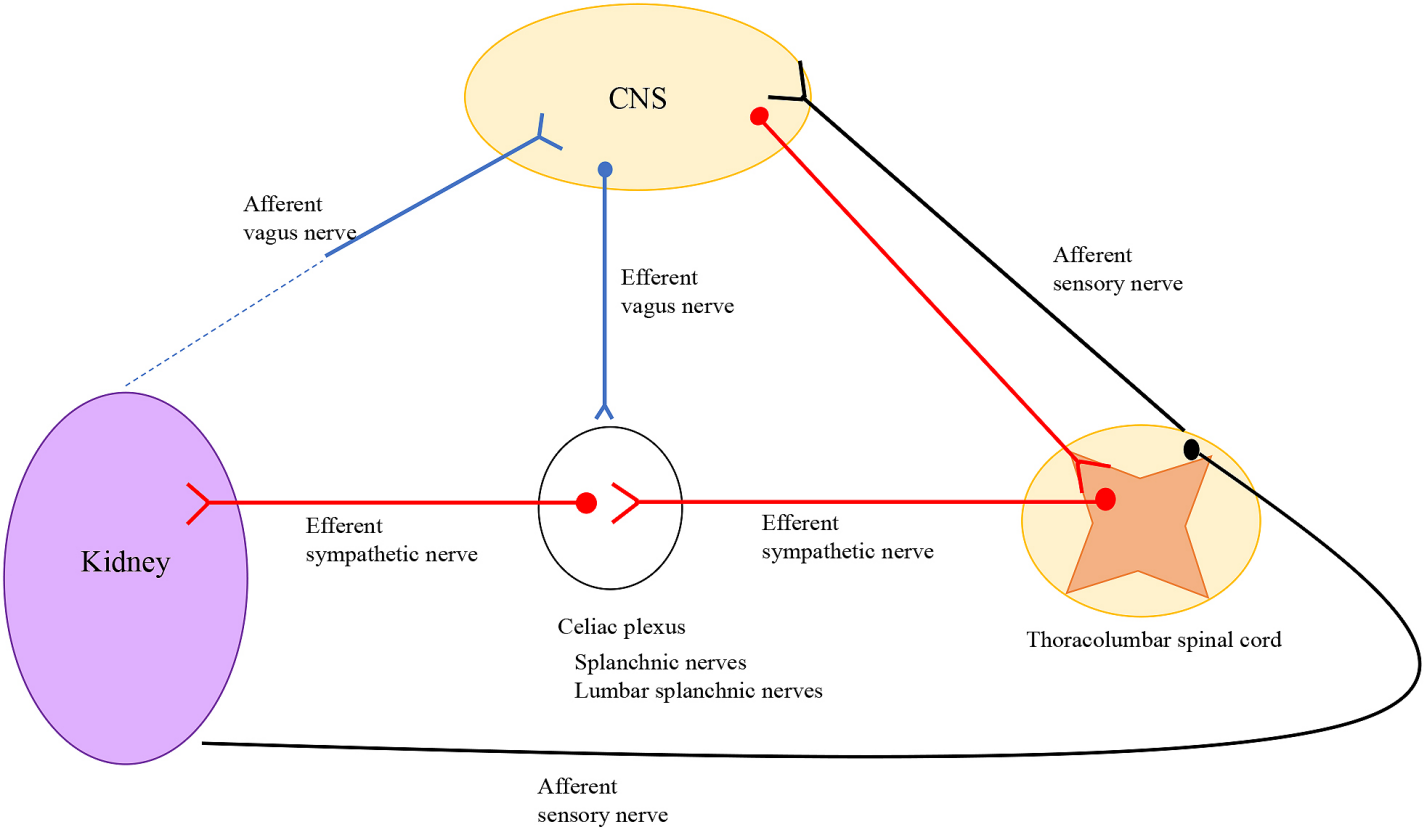
Innervation in Kidney. Sympathetic nerves and sensory nerves innervate in kidney. Innervation of parasympathetic nerves in kidney is controversial.

The kidney contains both efferent and afferent nerves (Figure 4). Extrinsic efferent sympathetic nerves are derived from the thoracic/lumbar spinal cord as preganglionic neurons, then synapse to postganglionic neurons in the paravertebral sympathetic chain ganglia and/or celiac, superior mesenteric, and aorticorenal ganglia ^(31)^. The sympathetic nerves innervate along with renal arteries and the intra-renal vasculature to reach the afferent arterioles, juxtaglomerular apparatus ^(32)^, tubules, and collecting ducts ^(33)^. Norepinephrine is released at the terminus of renal sympathetic nerves and acts on mainly arterioles and tubules. Sympathetic nerves play important roles in the kidney such as vasoconstriction and reduction of renal blood flow via α1_A_ adrenergic receptors on vascular smooth muscle cells ^(34)^, secretion of renin from the juxtaglomerular apparatus via β1 adrenergic receptors ^(35)^, and stimulation of tubular sodium reabsorption and indirectly water reabsorption via α1_B_ adrenergic receptors.

Although sympathetic nerves are widely distributed, sensory nerves are detected only in the pelvic region of the kidney and act as sensors of renal arterial/venous pressure and chemical contents ^(36)^. 

In contrast, there is little evidence of innervation of parasympathetic nerves (vagus nerve) in the kidney. Van Amsterdam et al. examined cadavers and found that parasympathetic fibers were present near the renal artery ^(37)^. However, these results were obtained by nitric oxide synthase antibody staining. Nitric oxide synthase is the only marker for parasympathetic innervation, but it is not specific and selective for parasympathetic innervation. In animal models, neurons synthesizing nitric oxide were found in a part of the renal sympathetic pathways. Maeda et al. detected ChAT-positive cells in rat tubules and collecting ducts by immunohistochemistry analysis using a ChAT antibody ^(38)^. This suggests the presence of acetylcholine in kidney but not direct innervation of parasympathetic nerves in the kidney.

## CAP and Kidney Injury

Increasing evidence, described below, suggests that CAP activation suppresses kidney injury; moreover, studies have revealed the mechanisms of CAP. There are several effects of CAP activation (Table 1), and recent studies have revealed an interaction between kidney injury and the inflammatory reflex.

**Table 1.** Applications of CAP Activator. There are several applications activating CAP. Electrical/transcutaneous vagus nerve stimulator are already used in the clinic. Abbreviations: VNS; vagus nerve stimulation

Modalities	Advantages	Disadvantages	Clinical application	Clinical trials Or pilot study
Ultrasound	Non-invasive	Uncertain mechanism		
Implantable device (Electrical stimulation)	Technically easy	Invasive Nonselective	Cluster headache Depression Migraine Functional disorder of intestine	Rheumatoid arthritis ^(38)^ Crohn’s disease ^(39)^
Transcutaneous VNS (Non-invasive VNS)				Epilepsy ^(61)^ Depression ^(62)^ Migraine ^(63), (64)^ Cluster headache ^(65)^
Optogenetic stimulation	Selective stimulation	Invasive Technically difficult		
Drugs (nicotine, GTS-21)	Easy clinical application	Off-target effects		

### 1. Drugs (acetylcholine receptor agonists)

To determine the mechanisms of CAP, nicotine (a nicotinic ACh receptor agonist) and GTS-21 (α7nAChR specific agonist) have been evaluated in pre-clinical settings. Several animal experiments have revealed that these AChR agonists have renoprotective effects. In 2008, Yeboah et al. reported that nicotine and GTS-21 attenuated bilateral IRI-induced kidney damage ^(39)^. Although pretreatment with these agonists reduced plasma creatinine elevation and tubular necrosis, delayed treatment (2 h post-reperfusion) did not improve kidney injury. Chatterjee et al. verified these results in another AKI model. In an LPS model, nicotine and GTS-21 attenuated renal leukocyte infiltration and histological damage ^(39)^. In a cisplatin-induced AKI model, daily GTS-21 injection reduced the levels of cisplatin-induced kidney injury markers ^(40)^. These agonists are easy to use but have non-specific and off-target effects, limiting their application in clinical settings.

### 2. Ultrasound

Gigliotti et al. first showed that ultrasound treatment attenuated IRI-induced renal inflammation and accordingly ameliorated kidney injury ^(41)^. Anesthetized mice received abdominal ultrasound treatment 24 h before IRI showed reduced plasma creatinine levels and tubular necrosis compared to those in sham-operated mice. Ultrasound-treated mice also showed reduced levels inflammatory cytokines (interleukin-6 and TNF-α) as well as infiltration of neutrophils and macrophages in the kidney. Interestingly, unilateral (right side) ultrasound treatment did not attenuate the increases in plasma creatinine. Moreover, the spleen size and weight of ultrasound-treated mice were reduced, suggesting that the spleen is involved in ultrasound-mediated kidney inflammation. Additionally, mice that underwent splenectomy exhibited no renoprotective effects. To identify the immune cells involved in these effects, Rag1 knockout mice, which lack functional B cell and T cells, were used. Although Rag1 knockout mice showed no ultrasound-modulated protection from IRI, reconstruction of Rag1 knockout mice with wild-type CD4^+^ T cells 10 days prior to ultrasound treatment restored ultrasound-mediated renoprotection. α7nAChR is also involved in these ultrasound-induced renoprotective effects. The same experiments were performed in α7nAChR receptor knockout mice, which did not present renoprotective effects. Next, bone marrow chimeras with wild-type and α7nAChR knockout mice were used to determine the origin of α7nAChR, which revealed that hematopoietic cells α7nAChRs are responsible for mitigating IRI-induced inflammation ^(42)^. The spleen and α7nAChR are crucial for ultrasound-mediated renal protection from IRI. However, the process of transmission of efferent signals to the spleen remains unknown, as the splenic nerve is adrenergic, whereas acetylcholine is important for CAP activation. Chemical splenectomy by injection of 6- hydroxydopamine 14 days before IRI eliminated the ultrasound-mediated renoprotective effects. Taken together, the splenic nerve is also important for ultrasound-mediated renal protection from IRI. These effects were also observed in mice with cecal ligation puncture-induced sepsis ^(15)^.

### 3. Vagus nerve stimulation

Considering that vagus nerve stimulation activates CAP, Inoue et al. examined whether vagus nerve stimulation protects against kidney injury induced by IRI ^(43)^. Electrical stimulus of the vagus nerve at 24 h before IRI attenuated plasma creatinine elevation, tubular necrosis, and plasma TNF elevation. This protective effect occurred 24-48 h before IRI, but when vagus nerve stimulation was applied 10 min prior to IRI, the effect disappeared. This suggests that various inflammatory reactions are involved in IRI. Splenectomy conducted seven days before vagus nerve stimulation and IRI annulled the renoprotective effects. However, adoptive transfer of splenocytes from vagus nerve stimulation-treated mice protected against IRI-induced AKI. Because α7nAChR-positive macrophages are important in CAP ^(26)^, α7nAChR knockout mice were evaluated to confirm the involvement of α7nAChR-positive macrophages. In vagus nerve stimulation-treated α7nAChR knockout mice, no protective effects against IRI-induced AKI were observed. Moreover, adoptive-transfer of splenocytes from vagus nerve stimulation-treated α7nAChR knockout mice did not show protective effects, although splenocytes from wild-type mice are known to exert these effects. Therefore, α7nAChR-positive macrophages play a crucial role in protecting against IRI in the kidneys. Flow cytometry analysis revealed that the number of macrophages infiltrating the kidney increased after IRI, but the difference between wild-type and α7nAChR knockout mice was not significant. However, there were differences in the phenotypes of flow-cytometry-sorted macrophages. The expression of Arg1, an M2 (anti-inflammatory) macrophage marker gene, was suppressed by IRI, and vagus nerve stimulation restored its expression. However, Arg1 expression remained suppressed in vagus nerve stimulated-α7nAChR knockout mice. To investigate the role of α7nAChR-positive peritoneal macrophages in CAP, adoptive transfer of nicotine-treated wild-type or α7nAChR knockout peritoneal macrophages was performed ^(44)^. Transfer of macrophages from wild-type mice exhibited renal protection from IRI, whereas transfer of macrophages from α7nAChR knockout mice did not. This suggests that α7nAChR in peritoneal macrophages is important for CAP activation. Next, to explore the downstream signaling of α7nAChR in macrophages, RNA-sequencing analysis was performed to evaluate the differences between wild-type and α7nAChR knockout mice. Among genes showing lower expression in α7nAChR knockout mice-derived peritoneal macrophages compared to wild-type-derived peritoneal macrophages, hairy and enhancer of split-1 (Hes1), a basic helix-loop-helix DNA-binding protein, was further evaluated. Vagus nerve stimulation and ultrasound induced Hes1 expression in peritoneal macrophages. *In vitro* experiments confirmed that Hes1 knockdown inhibited nicotine-induced TNF-α suppression, whereas Hes1 overexpression suppressed LPS-stimulated TNF-α induction in macrophages and induced the expression of M2 macrophage markers. Adoptive transfer of Hes1-overexpressing macrophage cell lines into wild-type mice suppressed kidney injury resulting from IRI. Taken together, Hes1 is an important signaling molecule which activates CAP in kidney injury and is a candidate pharmacological target for activating CAP. 

The authors next evaluated whether the afferent or efferent vagus nerve participates in CAP activation and renoprotection ^(43)^. Because the vagus nerve bundle contains both afferent and efferent fibers, and mice cannot survive bilateral vagotomy, selective vagus nerve stimulation is impossible. The right vagus nerve was blocked with local anesthesia while the proximal or distal end of the left vagus nerve was being stimulated. Interestingly, not only the left efferent stimulation, but also left afferent vagus nerve stimulation had renoprotective effects in IRI mice ^(43)^. This suggests that another mechanism is involved in protection, such as a vagosympathetic reflex or activation of the HPA axis ^(45)^.

### 4. Optogenetics

**Figure 5.**
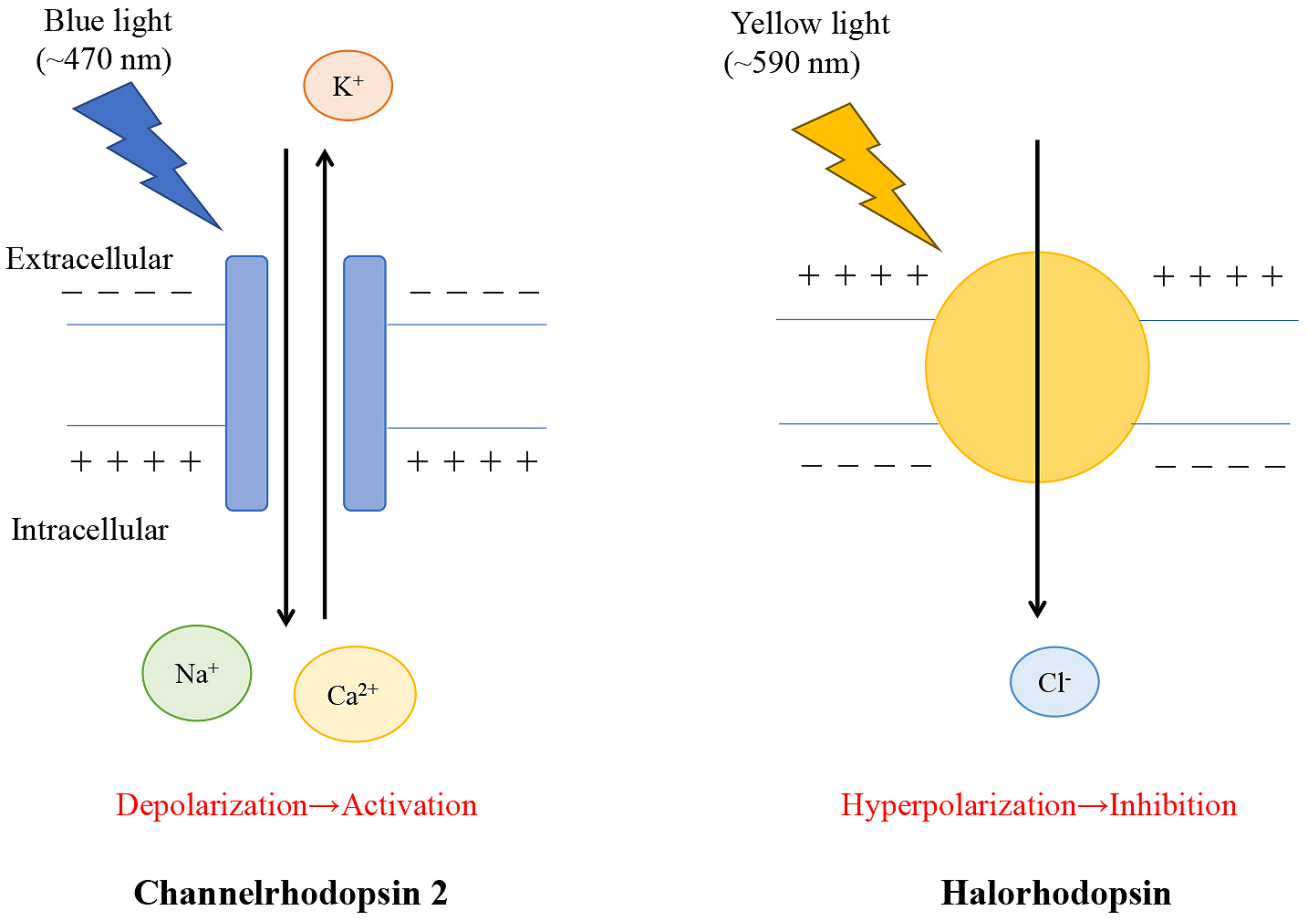
Optogenetics. Channelrhodopsin 2 or halorhodopsin are introduced genetically into specific cells or neurons for optogenetic stimulation. Blue light opens to channelrhodopsin 2. Then, cations flow into the specific neurons through the membrane. This evokes cell depolarization and eventually leads to neuronal firing. Yellow light opens to halorhodopsin, and one chloride ion enters the target cell through the membrane, hyperpolarizing the specific neuron. Selective cation channel (a chloride pump), resulting in excitation (inhibition) of the neurons.

Traditional methods, such as electrical stimulation, used to investigate neural networks in the central and peripheral nerves revealed CAP activation in kidney injury; however, there are limitations to these methods such as stimulating the specific target nerves at the proper time. Optogenetics is a novel technique that uses light to modulate molecular events via light-sensitive ion channels or pumps. This technique enables targetable control of gene-modified specific molecules. The discovery of channelrhodopsin-1 and channelrhodopsin-2 (ChR2) from algae was the origin of optogenetics ^(46), (47)^. ChR2 was initially applied to mammalian neurons ^(48)^, after which, this technique was developed mainly in the field of neuroscience to detect neuron activity at the millisecond scale in animals. This approach was named “optogenetics” in 2006 ^(49)^. ChR2 responds to blue light (450 ± 25 nm) to activate neurons via opening of non-selective cation channels. Halorhodopsin responds to yellow light (560 ± 27.5 nm) to inhibit neural activity via Cl^–^ influx (Figure 5) ^(50)^. These light-dependent-proteins are introduced into specific cells by the viral vector or Cre-LoxP systems for cell-specific activation. Furthermore, optogenetics revealed that the vagus nerve contains several subtypes. For example, in the lung, there are two populations of vagal afferent nerves (P2ry1, Npy2r), which project to the different brainstem targets and play opposing roles in breathing ^(51)^. Optogenetic stimulation of P2ry1 neurons attenuates breathing, whereas the stimulation of Npy2r neurons causes rapid and shallow breathing. Therefore, there are likely to be different subtypes of vagal nerves affecting CAP activation. Abe et al. performed optogenetics analysis and found that C1 neurons regulate CAP activation and attenuate IRI kidney injury ^(52)^. C1 neurons reside in the rostral and intermediate portions of the ventrolateral medulla and innervate broadly in the brain, including the dorsal motor nucleus of the vagus nerve, paraventricular nucleus of the hypothalamus, other regions of the brainstem, and paraganglionic neurons of the sympathetic and parasympathetic nerves ^(53)^. C1 neurons are glutamatergic and catecholaminergic neurons involved in various physiological responses such as hypoglycemia, the HPA axis, glucose homeostasis, thermogenesis, breathing, and blood pressure. They also play an important role in stress responses, including pain, hypoxia, infection, inflammation, hemorrhage, and hypoglycemia. Additionally, C1 neurons are activated by interleukin-1 and LPS, and C1 may be involved in the inflammatory reflex. 

Abe et al. focused on C1 neurons as targets of CAP. They confirmed that both restraint stress and optogenetic C1 neuron stimulation by blue light protected the kidneys from IRI injury, which involved the spleen, β2-adrenergic receptors, and α7nAChRs. In addition, restraint-stress-mediated renoprotective effects were attenuated by Gi-coupled designer receptors exclusively activated by designer drugs, which inhibit C1 neuron selectivity. Thus, they found that C1 neurons are involved in the restraint-stress-induced anti-inflammatory pathway. Notably, subdiaphragmatic vagal denervation and a corticosterone receptor blocker did not inhibit the C1 neuron-mediated protective effects. These results suggest that C1 neurons activate CAP not via the vagal nerve, but rather, through a different pathway, such as sympathetic nerves (adrenergic) or the HPA axis.

## Vagus Nerve Stimulator

Vagus nerve stimulation has been adapted clinically for treating refractory epilepsy and refractory depression. Over 100,000 vagus nerve stimulators have been implanted, indicating that these devices are safe and tolerable for clinical use ^(54)^．Based on CAP activation, the anti-inflammatory effects of vagus nerve stimulator have reported several inflammatory diseases, and pilot studies have also suggested these effects. In rheumatoid arthritis, 17 patients showed improvements in symptoms during vagus nerve stimulator treatment ^(55)^. In Crohn’s disease, vagus nerve stimulator improved biological parameters in seven patients at six-month follow up, with five patients showing endoscopic remission ^(56)^. Therapies for these diseases require the use of immunosuppressive agents, and vagus nerve stimulator is considered a feasible therapy option. Recent studies demonstrated the effectiveness of vagus nerve stimulator for treating pancreatitis ^(57)^, ileus, diabetes ^(58)^, hypertension ^(59)^, and heart failure ^(60)^. Moreover, transcutaneous vagus nerve stimulator, which uses noninvasive devices, was developed and shown to effective for treating epilepsy ^(61)^, depression ^(62)^, migraine ^(63), (64)^, and cluster headache ^(65)^ in preclinical studies.

## Conclusions

In this review, we summarize current knowledge regarding the interaction between neuroimmune interactions and kidney disease. To determine the mechanisms of CAP, further studies are needed. However, many pre-clinical studies have suggested that CAP activation mediates kidney injury. Vagus nerve stimulator has been applied for treating various diseases in clinical settings, and thus, proves to be a promising therapy for AKI. Additional studies are required to ensure its safe and effective clinical use.

## Article Information

### 

This article is based on the study, which received the Medical Research Encouragement Prize of The Japan Medical Association in 2019.

### Conflicts of Interest

None

### Sources of Funding

This work was supported by Grant-in-Aid for Research Activity start-up (JSPS KAKENHI grant number 18H06192 to TI), AMED (grant number JP19gm6210013h0001 to TI), MSD Life Science Foundation, Public Interest Incorporated Foundation (to TI), The Kidney Foundation (grant number JKFB18-3 to TI), The Salt Science Research Foundation (grant number 1919 to TI), Smoking Research Foundation (grant number 2019T006 to TI), Yukiko Ishibashi Foundation (to TI), The Mochida Memorial Foundation for Medical and Pharmaceutical Research (to TI), Takeda Science Foundation (to TI), Astellas Foundation for Research on Metabolic Disorders (to TI), and Suzuken Memorial Foundation (to TI).

### Acknowledgement

We would like to thank Editage (www.editage.com) for English language editing.

### Author Contributions

Yasuna Nakamura wrote the original manuscript. Tsuyoshi Inoue revised the manuscript.
